# Intra-Subject Variability in Pharmacokinetics and Pharmacodynamics of Basal Insulin at Two Single-Dose Levels: Findings from Euglycemic Glucose Clamp Bioequivalence Studies of Insulin Degludec

**DOI:** 10.3390/pharmaceutics18091051

**Published:** 2026-08-25

**Authors:** Hui Liu, Ting Li, Xinlei Chen, Hongling Yu, Yuchun Men, Huiwen Tan, Jiaqi Li, Yerong Yu

**Affiliations:** 1 Department of Endocrinology and Metabolism, West China Hospital of Sichuan University, Chengdu 610041, China; liuhui@wchscu.cn (H.L.); dym_9000@163.com (X.C.); hxyhl66@126.com (H.Y.); huiwent2016@scu.edu.cn (H.T.); jq_lee5477@163.com (J.L.); 2 Clinical Trial Center, West China Hospital of Sichuan University, Chengdu 610041, China; 18982101860@163.com; 3 Health Management Center, West China Hospital of Sichuan University, Chengdu 610041, China; endocrinelt520@163.com

**Keywords:** euglycemic clamp, healthy subjects, bioequivalence, intra-subject variability, basal insulin formulations, pharmacokinetics, pharmacodynamics, insulin degludec

## Abstract

**Background/Objectives**: Standard protocols for euglycemic clamp studies involving long-acting insulin formulations typically recommend a dosage range of 0.4~0.6 U/kg for a single dose. This investigation aimed to evaluate the bioequivalence of an insulin degludec (IDeg) biosimilar versus its reference product, while simultaneously analyzing intra-subject variability in pharmacokinetic (PK) and pharmacodynamic (PD) metrics at doses of 0.4 U/kg and 0.5 U/kg in healthy Chinese adults. **Methods**: This randomized, single-dose, crossover euglycemic clamp study involved 52 participants who received either 0.4 U/kg (Group A, *n* = 26) or 0.5 U/kg (Group B, *n* = 26) of both formulations. Key outcomes measured included AUC_IDeg,0–24h_, C_max,IDeg_, and AUC_GIR,0–24h_. Secondary endpoints encompassed AUC of IDeg or GIR at specified intervals and time-related metrics. Intra-subject variability (intra-CV) was assessed for PK/PD parameters. **Results**: All subjects completed the study without dropping out. Baseline demographics showed no significant disparities between groups. Bioequivalence criteria were satisfied for all PK/PD endpoints, with 90% confidence intervals (CIs) falling within the 0.80~1.25 range, with the exception of AUC_GIR,0–12h_ at the 0.4 U/kg dose (90% CI: 0.944~1.333). Notably, the 0.4 U/kg dose exhibited significantly higher intra-CV for AUC_GIR,0–24h_ (24.9% vs. 20.2%, *p* = 0.049) and AUC_GIR,0–12h_ (37.6% vs. 27.6%, *p* = 0.005) compared to the 0.5 U/kg dose. **Conclusions**: Applying a 0.5 U/kg dose reduced intra-subject PD variability and improved equivalence likelihood for secondary endpoints, indicating that a higher single dose might be optimal in bioequivalence study design.

## 1. Introduction

Insulin remains the cornerstone of diabetes mellitus management, yet the market is heavily consolidated, with three major global corporations controlling approximately 99% of the market value and 96% of the volume [1]. Consequently, insulin costs remain prohibitively high in many regions. Data from 11 countries indicate that the average annual cost per individual is US$35.40 in the public sector and US$95.71 in the private sector [2]. As of 2019, insulin availability in low- and middle-income countries ranged between 55% and 80% [3]. The introduction of biosimilar insulins presents a viable pathway to alleviate the financial burden of insulin therapy [4,5,6]. Regulatory bodies, including the US Food and Drug Administration (FDA) [7,8] and the European Medicines Agency (EMA) [9], mandate that the pharmacokinetic (PK) and pharmacodynamic (PD) equivalence of a biosimilar must be established against the reference product using the euglycemic clamp technique prior to market approval.

Selecting the appropriate insulin dose is pivotal for the success of euglycemic clamp studies. Historical data reveal a wide variance in insulin dosing for these studies, ranging from as low as 0.05 U/kg [10] to as high as 2.22 U/kg [11]. Suboptimal dosing can lead to methodological flaws; for instance, lower doses may elicit only minimal glucose infusion rate (GIR) responses [12]. Studies utilizing lower doses (e.g., 0.3 U/kg for insulin glargine) [13,14] have occasionally yielded irreproducible results, a phenomenon often attributed to elevated intra-subject variability. Furthermore, clinical observations suggest that subcutaneous insulin administration does not always produce a consistent metabolic response, even under controlled conditions. Moreover, few studies [15,16,17,18] have investigated variability in absorption profiles following subcutaneous injection, with most research focusing on differences among various insulin types. Even fewer studies [16,19] have examined variations in glucose-lowering efficacy. Consequently, there is a paucity of data regarding how varying insulin doses impact time-based patterns and their associated glucose-lowering effects, particularly for basal insulin formulations.

Euglycemic clamp studies typically employ a crossover design to assess bioequivalence. The required sample size is heavily influenced by the intra-subject variability of the endpoints [20,21]; minimal GIR responses can inflate the variability of PD parameters, necessitating larger sample sizes and increased resources. Conversely, high-dose insulin clamps present their own challenges, such as managing rapid blood glucose fluctuations [12]. Even a single episode of hypoglycemia [12] or insufficient suppression of endogenous insulin [22] can compromise the validity of the clamp.

While EMA guidance [9] suggests a dose range of 0.4~0.6 U/kg for long-acting insulins, direct data comparing the impact of specific dose selections (e.g., 0.4 vs. 0.5 U/kg) on intra-subject variability and bioequivalence outcomes remain scarce. To address this gap, the current study analyzes the PK/PD bioequivalence and intra-subject variability of insulin degludec (IDeg) at doses of 0.4 and 0.5 U/kg in healthy Chinese subjects following a single subcutaneous injection.

## 2. Materials and Methods

### 2.1. Subject and Study Design

Participants receiving a dose of 0.4 U/kg were assigned to Group A, while those administered 0.5 U/kg were placed in Group B. Eligibility criteria were strictly defined: individuals aged 18–45 years, with a body mass index (BMI) between 18 and 26 kg/m^2^, normal glucose tolerance (fasting glucose < 6.1 mmol/L and 2-h post-load glucose < 7.8 mmol/L), and no history of hypertension, diabetes mellitus, or substance abuse. Health status was verified through comprehensive medical history reviews, laboratory tests (hematology, biochemistry, urinalysis, coagulation), physical examinations, and electrocardiograms. Smokers (≥5 cigarettes/day) and pregnant or lactating women were excluded.

**Figure 1:**
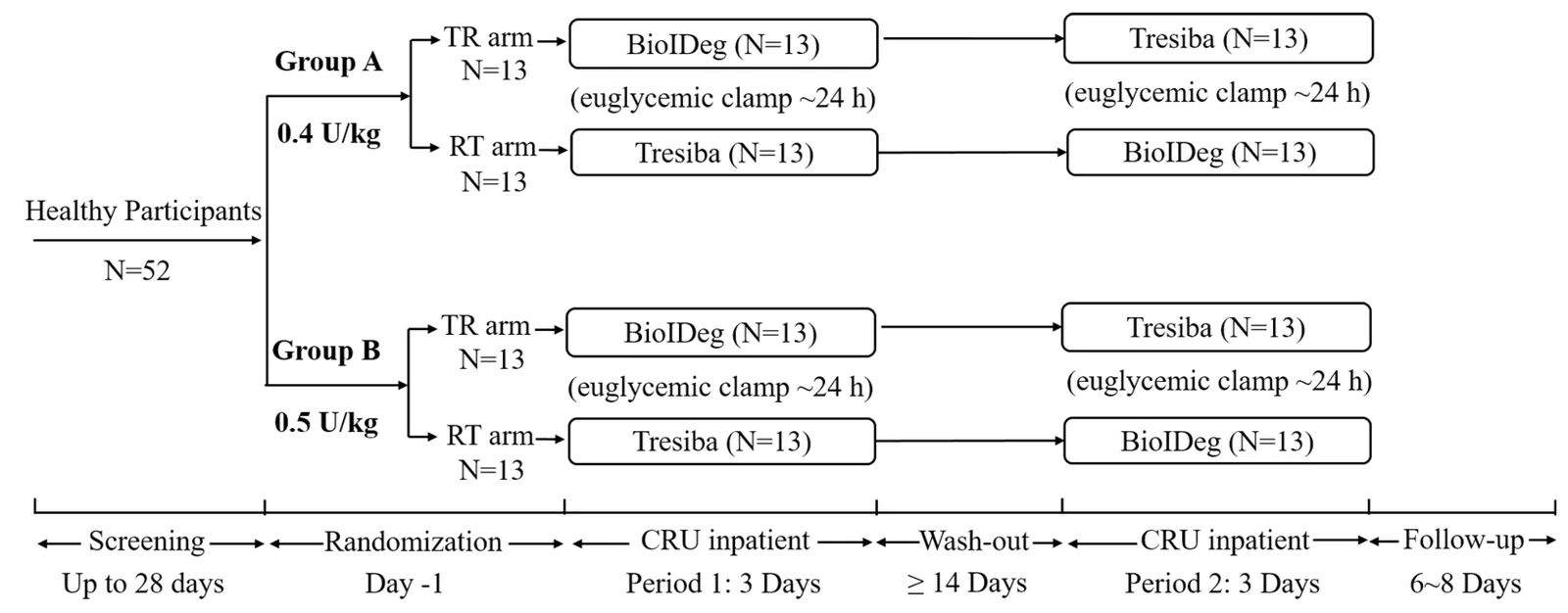
Study design/BioIDeg: biosimilar insulin degludec; CRU: clinical research unit.

The study protocol comprised a screening visit, two dosing periods separated by a washout interval of at least 14 days, and a follow-up visit (see Figure 1). On day 1, subjects were randomized 1:1 to either the TR or RT treatment sequence. They were admitted to the research unit at 18:00 the day prior to dosing. Following an overnight fast, each participant received a single subcutaneous injection of IDeg into the abdominal wall at the assigned dose (0.4 U/kg or 0.5 U/kg).

### 2.2. Euglycemic Clamp Procedure

On the morning of the dosing day, two venous catheters were inserted: one for blood sampling and another for the infusion of 20% dextrose. Baseline blood glucose (BG) was established as the mean of measurements taken at −30, −20, and −10 min pre-dose. Post-administration, BG was monitored every 10 min (0~8 h), every 20 min (8~16 h), and every 30 min (16~24 h) using a Biosen C_line glucose analyzer (EKF Diagnostics, Barleben, Germany). The 20% dextrose infusion was triggered when BG dropped by ≥0.28 mmol/L from baseline and was continuously titrated to maintain BG at a target level (0.28 mmol/L below baseline). The clamp was maintained for 24 h.

### 2.3. Blood Sampling and Bioanalysis for PK and C-Peptide

Blood samples (4 mL for PK and 2 mL for C-peptide) were collected at predefined intervals as previously described [23]. IDeg concentrations in plasma were quantified using a validated liquid chromatography-tandem mass spectrometry (LC-MS/MS) method with a quantification range of 0.25 to 50.0 ng/mL, a precision of ≤5.7%, and an inter-assay variation ranging from −0.3% to 2.0%. C-peptide levels in serum were measured using a qualified enzyme-linked immunosorbent assay (ELISA) method, which had a quantification range of 30 to 2400 pmol/L, an intra-assay variation of <5%, and an inter-assay variation of <9%.

### 2.4. Pharmacokinetic and Pharmacodynamic Estimates

Primary PK metrics included the area under the curve (AUC) from 0 to 24 h (AUC_IDeg,0–24h_) and peak concentration (C_max,IDeg_). Secondary PK parameters included AUC at various intervals (0–2 h, 12–24 h, 0–120 h), time to maximum concentration (T_max_), and half-life (t_1/2_). The primary PD endpoint was the AUC of the glucose infusion rate (GIR) over 24 h (AUC_GIR,0–24h_). Secondary PD parameters included AUC_GIR_ at specified intervals, maximum GIR (GIR_max_), and time to GIR_max_ (tGIR_max_). Calculations were performed using Phoenix WinNonlin (v8.1).

### 2.5. Sample Size and Statistical Analysis

Assuming a maximum intra-subject variation of 20.5%, a sample size of 26 was calculated to achieve 91% power at a 5% significance level using a two one-sided test procedure. This calculation was based on an expected true mean ratio of biosimilar IDeg (BioIDeg) to the reference product (Tresiba, Novo Nordisk, Bagsværd, Denmark) of 0.95, with equivalence limits set between 0.80 and 1.25. Primary PK and PD parameters were log-transformed and analyzed via a linear mixed model including treatment, sequence, and period as fixed effects. Bioequivalence was confirmed if the 90% CIs for the least squares geometric mean (LS- geometric mean) ratio fell within 80~125% [7,8,9]. Time-related parameters were assessed using the Wilcoxon signed-rank test. Clamp quality was evaluated using the coefficient of variation of BG (CVBG) and mean absolute relative deviation (MARD) [24]. The intra-subject variability (intra-CV) was calculated using the following equation: $intra\;CV\%=\sqrt{\left(exp(\sigma^{2})-1\right)}$ [18]. An *F*-test was used to compare the intra-CV between the two dose levels. Analyses were conducted using SPSS (v27.0), with statistical significance set at *p* < 0.05.

## 3. Results

### 3.1. Subject Disposition and Demographics

As illustrated in Figure 1, 52 participants were enrolled and equally distributed between Group A and Group B. All subjects completed the study. Table 1 confirms that there were no statistically significant differences between the groups regarding gender distribution (34.6% vs. 26.9% female, *p* = 0.764), age (26.5 ± 2.8 vs. 28.4 ± 6.1 years, *p* = 0.155), weight (60.5 ± 9.0 vs. 61.7 ± 9.0 kg, *p* = 0.618), height (166.7 ± 6.5 vs. 168.0 ± 8.8 cm, *p* = 0.526), or BMI (21.7 ± 2.0 vs. 21.8 ± 2.0 kg/m^2^, *p* = 0.811).

### 3.2. Euglycemic Clamp Quality Assessment

Table 2 indicates that baseline BG (Group A: 4.48 ± 0.27 vs. 4.44 ± 0.27 mmol/L; Group B: 4.65 ± 0.28 vs. 4.69 ± 0.32 mmol/L), target BG (Group A: 4.20 ± 0.27 vs. 4.16 ± 0.27 mmol/L; Group B: 4.37 ± 0.28 vs. 4.41 ± 0.32 mmol/L), and clamped BG levels (Group A: 4.22 ± 0.25 vs. 4.20 ± 0.25 mmol/L; Group B: 4.40 ± 0.27 vs. 4.42 ± 0.28 mmol/L) were comparable between the two formulations across both groups. In addition, the clamped BG levels were close to the target values. Similarly, comparable CVBG (Group A: 3.31 ± 0.71 vs. 3.61 ± 1.19; Group B: 3.15 ± 0.58 vs. 3.02 ± 0.61) and MARD (Group A: 2.77 ± 0.79 vs. 2.94 ± 0.80; Group B: 2.56 ± 0.51 vs. 2.51 ± 0.61) values were observed between the two drugs, with both measures remaining relatively low. Baseline C-peptide levels (Group A: 385 vs. 405 pmol/L; Group B: 380 vs. 364 pmol/L) were analogous between the two formulations across the groups, and a comparable reduction in C-peptide (Group A: 51.2% vs. 44.7%; Group B: 37.7% vs. 37.8%) was detected across treatments in both groups A and B. No significant increase in post-dose C-peptide (e.g., by a 0.5-fold increase from baseline) was observed.

### 3.3. PK/PD Parameters of IDeg at Two Dose Levels

**Figure 2:**
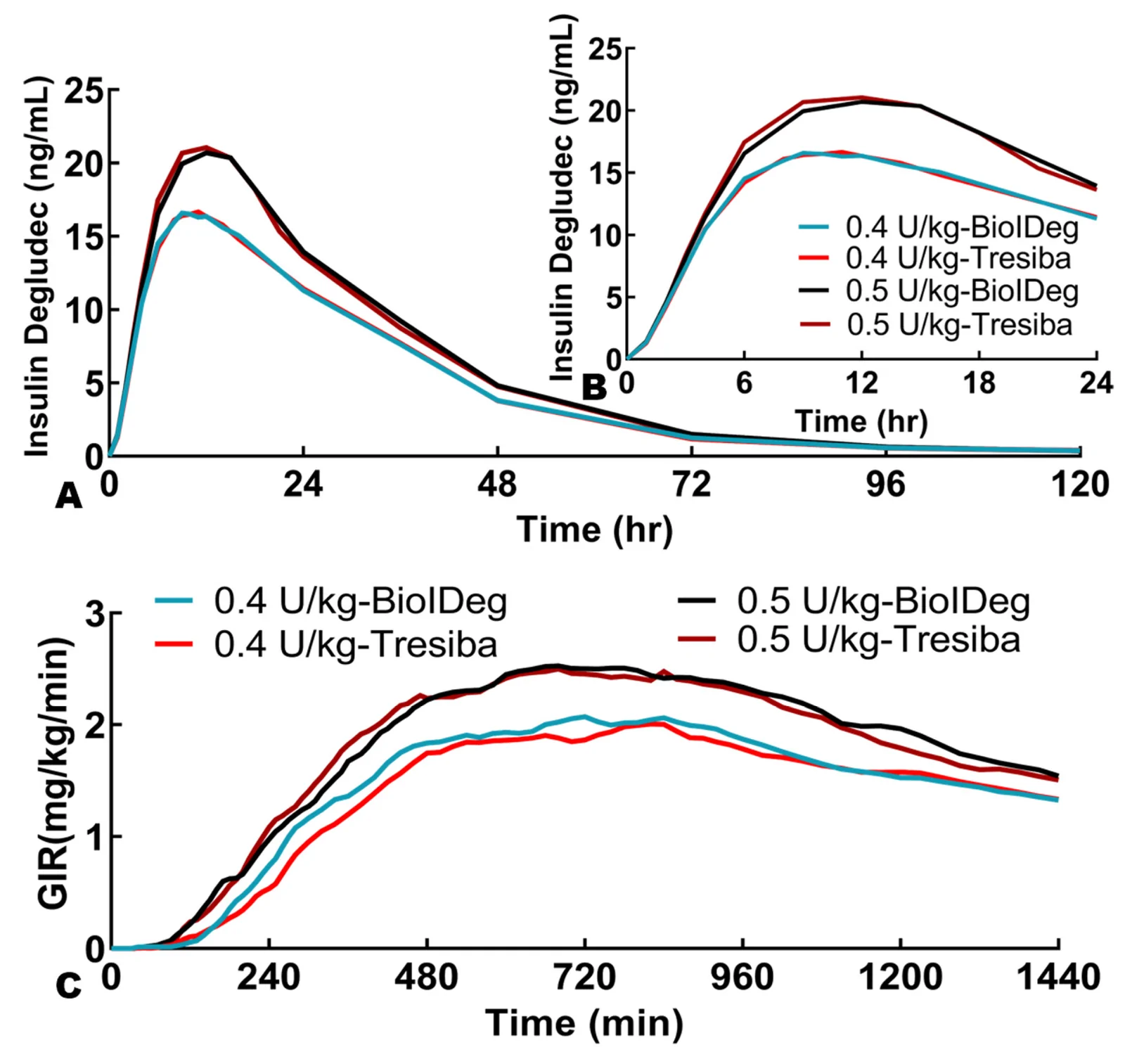
Time profiles of mean values of insulin degludec (IDeg) concentrations of two formulations at two dose levels (0.4 and 0.5 U/kg) from 0 to 120 h (**A**), and 0 to 24 h (**B**). Time profiles of mean values of glucose infusion rate (GIR) of two formulations at two dose levels (0.4 and 0.5 U/kg) from 0 to 24 h (**C**).

Figure 2A,B illustrate the PK profiles of IDeg. Following subcutaneous injection, both dose levels exhibited similar concentration-time curves across the two formulations. The LS-geometric mean ratios of BioIDeg to Tresiba for both primary and secondary PK endpoints were all close to 1, with a range of 0.981~1.003 (Table 3); moreover, the corresponding 90% CIs were entirely within the acceptable range of 0.80 to 1.25. No differences were detected in T_max_ (group A: 11.0 vs. 11.0 h; group B: 11.6 vs. 11.5 h) and t_1/2_ (group A: 14.8 vs. 13.5 h; group B: 16.1 vs. 15.5 h).

Figure 2C shows similar GIR-time profiles for the two formulations at 0.4 U/kg and 0.5 U/kg doses. For AUC_GIR 0–24h_, the LS-geometric mean ratios of BioIDeg to Tresiba were near 1 at both doses, with 90% CIs within the 0.80~1.25 equivalence range. Regarding the secondary pharmacodynamic parameter AUC_GIR,0–12h_, the 90% CI of the LS-geometric mean ratio was 0.944~1.333 at 0.4 U/kg (outside the equivalence range) but 0.856~1.132 at 0.5 U/kg (meeting equivalence criteria). The 90% CIs of the LS-geometric mean ratio for other PD parameters (AUC_GIR, 12–24h_ and GIR_max_) were within acceptable limits. No significant differences in tGIR_max_ (group A: 820 vs. 830 min; group B: 740 vs. 685 min) were observed between formulations at the same dose.

### 3.4. Comparisons of Intra-CV of PK/PD Parameters

As detailed in Table 4, intra-CV for PK parameters was largely comparable between formulations, with the only statistically significant difference found in AUC_IDeg,0–120h_ (*p* = 0.033). However, this absolute difference was clinically negligible (<5%). In contrast, PD parameters showed notable dose-dependent differences. The 0.4 U/kg dose was associated with significantly higher intra-CV for AUC_GIR,0–24h_ (24.9% vs. 20.2%, *p* = 0.049) and AUC_GIR,0–12h_ (37.6% vs. 27.6%, *p* = 0.005) compared to the 0.5 U/kg dose.

Based on the observed intra-CV for AUC_GIR, 0–24h_ (24.9% for 0.4 U/kg vs. 20.2% for 0.5 U/kg), a retrospective power analysis indicates that to achieve >90% power, a study using the 0.4 U/kg dose would require approximately 32 subjects, whereas the 0.5 U/kg dose would only require 24 subjects.

## 4. Discussion

This study evaluated the PK/PD similarity between a biosimilar IDeg formulation and its reference product in healthy Chinese participants, specifically focusing on the impact of dose selection (0.4 vs. 0.5 U/kg) on bioequivalence and intra-subject variability. The euglycemic clamp technique, recognized as the gold standard for PK/PD evaluation of insulin products [25], relies on high-quality performance to provide accurate estimates. The quality was confirmed by a mean CVBG below 5% and mean MARD under 3%, alongside sustained C-peptide suppression, ensuring reliable data for PK/PD assessment.

Insulin biosimilars play a vital role in improving patient access to cost-effective treatment options [4,5,6]. Local regulations require Phase I euglycemic clamp studies, with the EMA guidelines recommending a dosage range of 0.4~0.6 U/kg for long-acting insulin preparations [9]. The 90% CIs for the LS-geometric mean ratios for primary PK/PD endpoints were within the equivalence range of 0.80~1.25 at doses of 0.4 U/kg and 0.5 U/kg, demonstrating PK/PD similarity at both dose levels. Specifically, the 0.4 U/kg dose failed to demonstrate equivalence for AUC_GIR,0–12h_, whereas the 0.5 U/kg dose succeeded. Although no significant discrepancies were observed in bioequivalence evaluations between the two doses, a higher dose may enhance the likelihood of meeting equivalence criteria for secondary PD parameters. The upward trends in IDeg and GIR profiles at doses of 0.5 U/kg compared with those of 0.4 U/kg aligned with previous literature [26,27], though a shorter half-life (t_1/2_) was noted compared with previously reported values of 24~25 h [27,28], likely due to the single-dose design of this study. In the present work, C_max,IDeg_, T_max_, and t_1/2_ are used to characterize the PK profiles; however, these may not fully represent the terminal phase due to the ‘flip-flop phenomenon’. Flip-flop pharmacokinetics, common with extravascular drug administration, arises when absorption is slower than elimination. Overlooking this phenomenon can hinder the precise measurement and reliable interpretation of key PK parameters, such as underestimating the true elimination half-life and misclassifying drug disposition traits [29]. Given the scarcity of comprehensive intravenous (I.V.) PK data for IDeg, which directly reveals systemic elimination without absorption interference, IDeg’s subcutaneous PK profiles should be interpreted cautiously.

Intra-CV for most PK parameters was comparable, with statistical significance observed only in AUC_IDeg,0–120h_, considered clinically irrelevant due to its negligible impact on sample size estimation. Nonetheless, there was a notable increase in intra-CV for AUC_GIR,0–24h_, and AUC_GIR,0–12h_ in group A. Dose-dependent increases by 20%~50% were observed in AUC_GIR,0–12h_, AUC_GIR,0–24h_, AUC_GIR,12–24h_, and GIR_max_ at 0.5 U/kg dose compared to 0.4 U/kg dose, highlighting the dose’s influence on PD response parameters. Our observation that the lower dose failed to demonstrate equivalence for a secondary endpoint (AUC_GIR,0–12h_) underscores the concept that variability acts as a limiting factor, as previously reviewed by Vora and Heise [30]. The primary factors influencing variability in PD parameters include insulin absorption and metabolism in vivo, as well as insulin sensitivity [30,31]. This study observed a 19% reduction in the intra-CV of AUC_GIR,0–24h_ when switching IDeg dose from 0.4 U/kg to 0.5 U/kg, with comparable intra-CVs in AUC_IDeg,0–24h_ (7.8% vs. 8.0%). The observed reduction in intra-CV at the 0.5 U/kg dose can be rationalized by the insulin dose–GIR response principle. Similar to the formula, $CV=\frac{\sigma }{\mu }$, a smaller mean GIR response (μ) results in a larger CV for a given standard deviation of error. This phenomenon aligns with that lower insulin doses may elicit a diminished GIR response, magnifying the PD variability [12], and our findings align with this expectation as demonstrated by our data (intra-CV of AUC_GIR,0–24h_: 24.9% vs. 20.2%, *p* = 0.049). Among the PK/PD parameters, the highest variability was noted in AUC_0–12h_. After subcutaneous administration, IDeg dissolves into the bloodstream gradually, with absorption rates varying most across subjects during the initial 12 h. This variability in IDeg absorption was amplified by the differences in GIR response, especially at lower doses.

This single-dose euglycemic clamp study suggests that IDeg’s early absorption kinetics (0~12 h post-dose) may show dose dependence, with lower variability at higher doses (AUC_IDeg,0–12h_: 11.9% vs. 9.8%, *p* = 0.066), though not statistically significant—aligning with Haahr and Heise [28]. Beyond 12 h, IDeg levels stabilize, resulting in consistent PK/PD profiles, as indicated by the reduced intra-CV of AUC_IDeg,12–24h_ and AUC_GIR,12–24h_. These results imply higher insulin doses could decrease early-phase PD variability and improve the feasibility of bioequivalence assessments through single-dose euglycemic clamp studies.

While both doses showed bioequivalence for the primary endpoint, choosing the 0.5 U/kg dose has broader implications. To ensure over 90% power for detecting bioequivalence, sample size correlates with the square of the intra-CV. With AUC_GIR,0–24h_ intra-CV values of 24.9% (0.4 U/kg) versus 20.2% (0.5 U/kg), retrospective power analysis indicates a 0.4 U/kg study would need roughly 30% more participants to match the 0.5 U/kg dose’s statistical power. This increases operational complexity, costs, and participant burden. Thus, selecting 0.5 U/kg is not just a minor enhancement but a strategic optimization improving economic efficiency.

This study has several limitations: (1) Sample size calculations used an assumed maximum intra-CV of 20.5%, but the observed maximum intra-CV for the primary PD parameter was 24.9%; a larger sample size would improve statistical power. (2) Only two dose levels were tested; future research should assess additional doses (e.g., 0.6 U/kg). (3) Results apply only to euglycemic clamps with a single dose. For steady-state euglycemic clamps, we hypothesize that dosage selection does not significantly affect intra-CV of PK/PD parameters due to stable insulin levels under those conditions; this requires verification in future studies. (4) The study involved only healthy Chinese volunteers, despite IDeg being mainly prescribed to individuals with diabetes. Research shows type 1 diabetes duration does not significantly affect IDeg’s PK [32]. Thus, PK profiles in healthy volunteers may reflect those in the diabetic population, as the drug maintains consistent release irrespective of underlying metabolic or tissue pathology. However, recent clamp studies show BMI and adiposity correlate negatively with GIR [33,34], suggesting that healthy volunteer data may overestimate the glucose-lowering potency in obese type 2 diabetics or type 1 diabetics with insulin resistance. Some results may not apply to diabetic patients.

## 5. Conclusions

In conclusion, our study provides empirical evidence that a higher single dose is optimal for euglycemic clamp studies assessing basal insulin analogs in healthy Chinese subjects. Compared with 0.4 U/kg, the 0.5 U/kg dose not only ensures bioequivalence but also significantly reduces intra-subject pharmacodynamic variability. This finding has direct implications for the design of future bioequivalence trials, suggesting that adopting a relatively higher dose within the EMA-recommended range can enhance the robustness of PD assessments and increase the likelihood of trial success.

## Figures and Tables

**Table 1:** Subject disposition and demographic information of groups A and B.

	Group A (0.4 U/kg)	Group B (0.5 U/kg)	*p*
Number (n, female proportion)	26, 34.6%	26, 26.9%	0.764
Age (year) ^a^	26.5 ± 2.8	28.4 ± 6.1	0.155
Weight (kg) ^a^	60.5 ± 9.0	61.7 ± 9.0	0.618
Height (cm) ^a^	166.7 ± 6.5	168.0 ± 8.8	0.526
BMI (kg/m^2^) ^a^	21.7 ± 2.0	21.8 ± 2.0	0.811
TR treatment arm (n, %)	13, 50%	13, 50%	1.000

^a^ Mean ± standard deviation. BMI: body mass index.

**Table 2:** Clamp information of groups A and B.

	Group A (0.4 U/kg)			Group B (0.5 U/kg)		
	BioIDeg	Tresiba	*p*	BioIDeg	Tresiba	*p*
Basal BG (mmol/L) ^a^	4.48 ± 0.27	4.44 ± 0.27	0.583	4.65 ± 0.28	4.69 ± 0.32	0.634
Target BG (mmol/L) ^a^	4.20 ± 0.27	4.16 ± 0.27	0.586	4.37 ± 0.28	4.41 ± 0.32	0.634
‘Clamped’ BG (mmol/L) ^a^	4.22 ± 0.25	4.20 ± 0.25	0.692	4.40 ± 0.27	4.42 ± 0.28	0.739
CVBG (%) ^a^	3.31 ± 0.71	3.61 ± 1.19	0.277	3.15 ± 0.58	3.02 ± 0.61	0.750
MARD (%) ^a^	2.77 ± 0.79	2.94 ± 0.80	0.440	2.56 ± 0.51	2.51 ± 0.61	0.405
Basal C-peptide (pmol/L) ^b^	385 (134)	405 (131)	0.953	380 (260)	364 (216)	0.831
Post-dose C-peptide (pmol/L) ^b^	187 (118)	194 (80)	0.552	204 (157)	219 (209)	0.761
C-peptide reduction (%) ^b^	51.2 (18.7)	44.7 (17.1)	0.211	37.7 (20.9)	37.8 (15.8)	0.959

^a^ Mean ± standard deviation; ^b^ median (interquartile range). BG, blood glucose; CVBG, coefficient of variation of BG; MARD, mean absolute relative deviation from target; BioIDeg, Biosimilar insulin degludec.

**Table 3:** Pharmacokinetic and pharmacodynamic parameters following a single dose of 0.4 (group A) or 0.5 U/kg (group B) of insulin degludec.

	Group A (Geometric Mean with CV) (0.4 U/kg)			Group B (Geometric Mean with CV) (0.5 U/kg)		
	BioIDeg	Tresiba	LS-Mean Ratio (90% CI)	BioIDeg	Tresiba	LS-Mean Ratio (90% CI)
AUC_IDeg,0–24h_ (ng/mL × h)	301 (21.4%)	303 (16.0%)	0.993 (0.956, 1.031)	372 (19.4%)	377 (17.4%)	0.988 (0.953, 1.024)
AUC_IDeg,0–12h_ (ng/mL × h)	134 (31.0%)	135 (28.6%)	0.994 (0.940, 1.051)	159 (24.2%)	164 (25.2%)	0.969 (0.926, 1.014)
AUC_IDeg,12–24h_ (ng/mL × h)	165 (19.2%)	166 (13.1%)	0.993 (0.958, 1.030)	212 (18.2%)	211 (15.5%)	1.006 (0.969, 1.044)
AUC_IDeg,0–120h_ (ng/mL × h)	564 (19.4%)	562 (18.2%)	1.003 (0.986, 1.021)	710 (12.0%)	700 (11.2%)	1.013 (0.992, 1.035)
C_IDeg,max_ (ng/mL)	17.1 (25.8%)	17.5 (22.5%)	0.981 (0.940, 1.023)	21.3 (20.6%)	21.8 (18.1%)	0.979 (0.936, 1.024)
T_max_ (h)	11.0 (26.3%) ^a^	11.0 (22.5%) ^a^	0.00 (−1.00, 2.00) ^b^	11.6 (24.2%)	11.5 (33.2%)	0.00 (0.00, 0.00) ^b^
t_1/2_ (h)	14.8 (24.8%) ^a^	13.5 (25.5%) ^a^	0.43 (−2.25, 3.51) ^b^	16.1 (22.1%)	15.5 (26.9%)	1.28 (−2.37, 2.20) ^b^
AUC_GIR,0–24h_ (mg/kg)	1927 (41.2%)	1843 (40.0%)	1.046 (0.931, 1.175)	2443 (31.8%)	2393 (33.1%)	1.021 (0.924, 1.128)
AUC_GIR,0–12h_ (mg/kg)	734.4 (63.2%)	654.7 (59.2%)	1.122 (0.944, 1.333)	971 (39.0%)	986 (39.6%)	0.985 (0.856, 1.132)
AUC_GIR,12–24h_ (mg/kg)	1173 (29.7%)	1158 (30.6%)	1.012 (0.919, 1.115)	1448 (30.2%)	1392 (30.5%)	1.040 (0.952, 1.137)
GIR_max_ (mg/kg/min)	2.13 (48.2%)	2.07 (44.7%)	1.026 (0.927, 1.135)	2.75 (30.8%)	2.66 (38.2%)	1.034 (0.932, 1.146)
tGIR_max_ (min)	820 (22.9%) ^a^	830 (48.2%) ^a^	17.5 (0.0, 80.0) ^b^	740 (33.2%)	685 (24.5%)	120 (−80.0, 180) ^b^

^a^ Median (CV); ^b^ The difference between the medians with the 95% CI; AUC, area under curve; GIR, glucose infusion rate; BioIDeg, biosimilar insulin degludec; LS-mean, least square mean; CI, confidence interval.

**Table 4:** The intra-subject CV of pharmacokinetic and pharmacodynamic parameters.

	Group A (0.4 U/kg)	Group B (0.5 U/kg)	*p*
AUC_IDeg,0–24h_ (ng/mL × h)	8.0%	7.8%	0.791
AUC_IDeg,0–12h_ (ng/mL × h)	11.9%	9.8%	0.066
AUC_IDeg,12–24h_ (ng/mL × h)	7.7%	8.0%	0.684
AUC_IDeg,0–120h_ (ng/mL × h)	3.6%	4.5%	0.033
C_IDeg,max_ (ng/mL)	9.0%	9.6%	0.492
AUC_GIR,0–24h_ (mg/kg)	24.9%	20.2%	0.049
AUC_GIR,0–12h_ (mg/kg)	37.6%	27.6%	0.005
AUC_GIR,12–24h_ (mg/kg)	20.6%	18.7%	0.355
GIR_max_ (mg/kg/min)	21.6%	21.4%	0.916

AUC, area under the curve; IDeg, insulin degludec; GIR, glucose infusion rate. Participants in Group A received 0.4 U/kg of IDeg, while those in Group B received 0.5 U/kg.

## Data Availability

The data presented in this study are available on request from the corresponding author.
